# Autophagy in Cancer Progression and Therapeutics

**DOI:** 10.3390/ijms24097973

**Published:** 2023-04-28

**Authors:** Kamilla Kantserova, Ilya Ulasov

**Affiliations:** Group of Experimental Biotherapy and Diagnostics, Institute for Regenerative Medicine, World-Class Research Centre “Digital Biodesign and Personalized Healthcare”, I.M. Sechenov First Moscow State Medical University, 119991 Moscow, Russia; kam11_03@mail.ru

Autophagy is a catabolic process that is necessary for cellular homeostasis maintenance. Autophagy occurs due to stressful conditions such as nutrient deprivation, hypoxia, and DNA damage, or upon exposure of cancer cells to chemotherapy, which subsequently leads to the creation of autophagosomes that consume damaged organelles and long-lived proteins. Thereafter, autophagosomes move to the lysosome to form an autolysosome and recycle or remove dysfunctional cytosolic cargoes. In addition, even wild-type adenoviruses or conditionally replicating adenoviruses (CRAds) can trigger autophagy to maintain the infected cells’ viability for delivery of viral DNA and expression of adenoviral structural proteins [1]. Subsequently, infected cells die, and the viral progeny spreads to neighboring cells to continue the infection cycle [2].Autophagy is thus an essential process preventing normally functioning cells from becoming mutated or deregulated. In spite of this, abnormal autophagy is connected with various diseases, including cardio-related diseases, neurodegenerative diseases, and especially cancer [3]. Unfortunately, it is still unclear why autophagy has a controversial role as a cytoprotective mechanism through the elimination of toxic unfolded proteins or oncogenic protein substrates that is important for homeostasis. On the one hand, autophagy promotes tumor growth by meeting the biosynthetic needs of highly proliferative cancer cells and providing resistance to anticancer drugs. On the other hand, autophagy suppresses tumorigenesis in its early stages through the elimination of damaged organelles and cells [4]. This Special Issue summarizes recent advances made in understanding autophagy in cancer progression and therapeutics.

Chemotherapy often induces a number of cellular responses, such as autophagy, apoptosis, and senescence (Figure 1) [5]. One of the most effective chemotherapeutic agents used in the treatment of various cancer types is doxorubicin (DOX). However, DOX may cause the development of cardiotoxicity, and so its use is limited. The accumulation of mitochondrial reactive oxygen species is directly linked to the development of DOX cardiomyopathy [6]. Investigations into the role of autophagy in the heart have shown that a lack of the protein necessary for autolysosome formation (lysosome-associated membrane protein 2) leads to accumulation of autophagic vacuoles, dysregulation of protein degradation, and the development of cardiomyopathy [7,8]. On the other hand, an overexpression of autophagy results in cardiac dysfunction [9]. Unfortunately, the role of autophagy in DOX cardiotoxicity remains unclear. Nevertheless, Ryan et al. established that the maintenance of basal autophagy signaling in DOX-treated rats prevents oxidative damage to mitochondria, while a decrease in autophagosome formation below baseline disrupts the redox balance in the heart [10]. This clinical trial highlights the importance of time course studies in the development of strategies to prevent DOX cardiac dysfunction.

The importance of treating patients in a time-dependent manner is also observed in another investigation wherein chronic myelogenous leukemia cells were treated by Pyrimethamine (Pyri) [11]. It was established that autophagy activation correlates with time and concentration upon exposure to Pyri. Additionally, it was noticed that apoptosis increases because of drug concentration but is independent of time. Thus, this study suggested that Pyri can induce the expression of autophagy and apoptosis markers and lead to the regulation of different forms of cell death by causing the suppression of the oncogenic transcription factor STAT5 (signal transducer and activator of transcription 5). The recent studies of autophagy’s role in glioblastoma therapy have also changed the question of “how to modulate autophagy” to “when to modulate autophagy” [12]. The ability of glioblastoma cells to avoid cell death in the presence of chemotherapy is due to autophagy. Despite this, it is also known that an induction of overexpression of autophagy in glioblastoma can lead to cell death that functions synergistically with apoptosis. By focusing on the level of autophagy flux, a therapeutic strategy could be designed. However, elevated basal autophagic flux cannot always be detected under autophagy induction. For example, evidence from clinical trials suggests that the activity of the autophagy pathway is not raised in acute myeloid leukemia (AML) cell lines that are stably resistant (AraC-Res) to cytarabine (AraC) treatment, while AraC-sensitive cells increase the level of autophagy during AraC treatment [13]. AraC-Res cells cannot be re-sensitized to AraC using autophagy inhibitors; in spite of this, inhibition of autophagy in AraC sensitive cells can increase the cytotoxic effect of AraC treatment. Nevertheless, even the highest level of cell death during cotreatment is lower than the sum of each compound alone. This indicates that there are no additive cytotoxic effects of combinational treatment.

Autophagy plays a crucial role in cell survival, differentiation, metabolism, aging, and cell death. Cancer cells also need autophagy, particularly to meet biosynthetic needs. Autophagy represents a dual role in tumor suppression and progression. Both high and low levels of autophagy can have different effects on different types and stages of cancer. From all of the above, we can conclude that a wide range of factors lead to clinical outcomes that are not always expected. The targeting of such a controversial process as autophagy is extremely important to establish a gold standard for anticancer therapy.

## Figures and Tables

**Figure 1:**
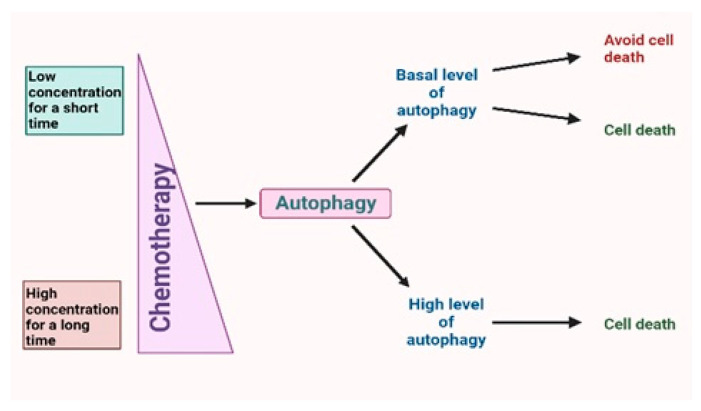
Chemotherapy leads to activation of autophagy, which can be divided into basal autophagy flax and high autophagy flax. Depending on the tumor type and stage basal level, autophagy can induce both cell death and, in most cases, resistance to anticancer drugs. In contrast, a time-dependent overexpression of autophagy caused by a high concentration of chemotherapy drugs leads mostly to cell death and tumor suppression. Thus, concentration and time of action of the drug can regulate autophagy flax.
